# Stakeholder perspectives on cooperation in the clinical and nonclinical health technology assessment domains

**DOI:** 10.1017/S0266462323000077

**Published:** 2023-05-22

**Authors:** Gina Zimmermann, Sandra Michelmore, Mickaël Hiligsmann

**Affiliations:** 1 Faculty of Health, Medicine and Life Sciences, Maastricht University, Maastricht, The Netherlands; 2 UCB Pharma Ltd., Slough, UK; 3 Department of Health Services Research, CAPHRI Care and Public Health Research Institute, Maastricht University, Maastricht, The Netherlands

**Keywords:** European Union, collaboration, health technology assessment, pharmaceutical technology, qualitative

## Abstract

**Objectives:**

The aim of this study was to deliver insights from multiple stakeholders into actual and future collaboration for health technology assessment (HTA) in general and in oncology in particular.

**Methods:**

Eighteen semi-structured interviews were conducted with experts from European HTA bodies (HTAbs), former board members of the European Network for Health Technology Assessment (EUnetHTA), and representatives from the pharmaceutical industry, a regulatory agency, academia, and patient organizations. The stakeholders were asked about their support of the EUnetHTA’s intent, about the general strengths and challenges of the EUnetHTA and its Joint Action 3 (JA 3), the strengths and challenges of the clinically oriented HTA collaboration in oncology during JA 3 across the technology life cycle, about future challenges to HTA in oncology with consequences for collaboration, and about collaboration in the economic domains of HTA. The transcribed interviews were analyzed qualitatively.

**Results:**

The participants perceived the intention and work quality of the EUnetHTA as positive. The experts described methodological, procedural, and capacity challenges in early dialogues (EDs) and rapid relative effectiveness assessments (REAs) meant to analyze clinical effectiveness in oncology. The majority attached increasing importance to collaboration in the future to cope with the uncertainty of HTA. Several stakeholders also proposed the incorporation of joint postlaunch evidence generation (PLEG) activities. Some gave sporadic suggestions for voluntary nonclinical collaboration as well.

**Conclusion:**

Stakeholders’ continued readiness to discuss the remaining challenges to and sufficient resources for implementing HTA regulation, as well as further cooperative expansion along the technology life cycle, are necessary for improved HTA collaboration in Europe.

## Background

The European Network for Health Technology Assessment (EUnetHTA) is a collaboration between network members and external partners that promotes the health technology assessment (HTA) of approved drugs, with the goal of driving efficient HTA collaboration (1). HTA is a multidisciplinary process that uses explicit methods to determine the value of a specific health technology at different points in its life cycle (2). Since its inception in 2006, the EUnetHTA has carried out three multiannual joint actions (JAs) to develop HTA collaboration.

Among its many projects, the initiative members created the HTA Core Model® as a fundamental methodological framework for producing and sharing HTA information (3). The model can be divided into clinical and nonclinical domains (3). Clinical domains include relative clinical effectiveness, defined as the extent to which a drug does more good than harm compared to one or more alternative interventions in achieving the desired results when provided under typical healthcare practice (3). Relative safety, the current technology, and the technical characteristics of the examined drug are also part of the model’s clinical elements (3). The EUnetHTA’s collaboration in JA 3 (2016–2021) focused on these clinical aspects, which led to horizon scanning (HS) activities that identify, select, and prioritize drugs for rapid relative effectiveness assessments (REAs) (4). In addition, in a candidate drug’s pre-licensing phase, the initiative conducted early dialogues (EDs) to enable confidential exchange between the industry, multiple HTA bodies (HTAbs), and, where applicable, the European Medicines Agency (EMA) to allow for the integration of HTA requirements into study designs and the generation of evidence (4). Furthermore, the EUnetHTA made early assessments of drugs’ clinical evidence compared to the standard of care by performing rapid REAs as joint clinical assessments (JCAs) through an examination of all clinical domains in the HTA Core Model® (3;4). Scattered pilot projects for specific products or registries for additional data and evidence collection were also carried out in the post-licensing phase to complement the evidence already generated (4). Furthermore, the EUnetHTA developed HTA-related templates and guidelines to strengthen the applicability of the initiative’s consolidated expertise (1).

In contrast, the nonclinical domains of the HTA Core Model® include costs and economic evaluation, ethical analysis, organizational aspects, and social or legal affairs (3). Although EUnetHTA-level cooperation among HTAbs has focused on the clinical domains of the HTA Core Model®, there are some other forms of collaboration on cost and economic evaluation between individual states. Participating countries in these initiatives are regionally close and use similar economic evaluation approaches. Specifically, the Northern Europe-based initiative between Finland, Norway, and Sweden, FINOSE, has started to perform several joint incremental cost-effectiveness ratio calculations to inform its collective price negotiations (5). The collaboration between Belgium, the Netherlands, Luxemburg, Austria, and Ireland, called BeNeLuxA, has likewise completed joint economic evaluations (6). However, recorded perceptions of collaboration in economic areas are rare.

Oncology faces a severe burden of disease and increasing challenges in the effective generation of evidence (7). Case-study-based policy recommendations demand collaboration among HTAbs, regulators, and patient organizations to make well-informed and faster decisions to assess and approve oncological innovations (7). The EUnetHTA has already included assessments for oncology in its activities. During JA 3, for instance, it conducted multiple EDs and five rapid REAs on this topic (8). It also performed two pilots for postlaunch evidence generation (PLEG) in oncology, one product- and one registry-specific (9).

In 2022, a new EU legal HTA framework (10) came into force, based on projects conducted during the EUnetHTA’s three JAs. The framework commits to a defined extent of cooperation in the clinical part of HTA, with its implementation set to follow a multi-stage procedure starting in 2025 (10). Oncology drugs will also play an important role from the beginning of the new framework’s implementation, as along with medicinal products for advanced therapy, they will be the first products with mandatory JCAs, as of 2025 (10).

This study aimed to provide expert insights into HTA cooperation in clinical and nonclinical domains and in oncology.

## Methods

Semi-structured interviews with experts of HTA in the oncology field were conducted to garner professional views of the strengths and challenges in the EUnetHTA’s collaboration. Nine questions were asked that generally referred to the strengths and challenges in clinically oriented HTA collaboration in oncology across the technology life cycle, along with opportunities for future economic collaboration. Each researcher approved the questions, with their final forms depicted in Table 1.

**Table 1.** Interview questions

Topic	Question
The General Strengths and Challenges of the EUnetHTA Initiative and JA 3	1) Likert scale question: I regard the intent of the EUnetHTA initiative as positive^a^ 2) Likert scale question: I regard the outcome of JA 3 as positive^a^ 3) What is your view on the general strengths and challenges of JA 3?
The Strengths and Challenges in the Clinically Oriented HTA Collaboration in Oncology during JA 3	4) Please provide your perspective on the strengths and challenges of each of these collaboration areas when assessing oncology drugs during JA 3 -HS -ED -Rapid REA -PLEG
The Future of HTA in Oncology	5) In your perspective, what are the expected main challenges for HTA in oncology? 6) Based on question 5, what consequences do you foresee for HTA collaboration in oncology?
The Potential of Voluntary Cooperation in Nonclinical Domains	7) What is your perspective on voluntary collaboration in oncology in the nonclinical aspects of HTA? 8) Likert scale question: I regard the clustering of individual EUnetHTA efforts into concepts of added value and cost-effectiveness/cost utility as feasible for voluntary cooperation^a^ 9) Based on question 8, what is your view of the opportunities and challenges of clustering into the concepts of added value and cost-effectiveness/cost utility for voluntary cooperation?

a On a scale of 1 (full refusal of the statement) to 5 (full agreement with the statement).

ED, early dialogue; EUnetHTA, European Network for Health Technology Assessment; HTA, health technology assessment; HS, horizon scanning; JA 3, Joint Action 3; PLEG, postlaunch evidence generation; rapid REA, rapid relative effectiveness assessment.

In total, 18 interviewees were selected. The experts were mainly identified through the lists of participants from EUnetHTA meetings or participants in the virtual EUnetHTA Forum in April 2021. Contacts provided by an internship organization and the researchers’ own networks led to further detection of participants. The experts first received an invitation letter via email, outlining the study’s background and scope. Before the interview, their written informed consent was obtained for collecting data including audio recordings, transcriptions, and pseudonymized data analysis. The interviews were conducted with Microsoft Teams in May and June 2021 and transcribed thereafter. Then, the resulting documents were sent back to the experts for editing and confirmation of their statements. Subsequently, the transcripts were evaluated using Mayring’s qualitative content analysis (11). Main categories were derived from the questions’ topics, and subcategories emerged inductively from the answers.

Ethical approval was obtained from the ethics officers of Maastricht University, and the research was classified as a low-risk project (FHML/HPIM/2021.036). At the same time, the confidentiality of the interview data and personal anonymity of the respondents were ensured.

## Results

The interviewees were categorized into four groups. The first group consisted of current or former senior board members of the EUnetHTA initiative (*n* = 2). The second group (*n* = 6) included representatives from HTAbs affiliated variously with the Netherlands, Germany, Finland, Sweden, Denmark, and Norway. The third group contained experts from the pharmaceutical industry (*n* = 2). Last, the mixed fourth group consisted of academic experts or consultants (*n* = 5), representatives from patient organizations (*n* = 2), and an expert from a regulatory agency (*n* = 1).

The following sections present the narrative of the main interview findings, while the number and the type of stakeholder behind it indicate how many and which participants in total have made a certain statement. Table 2 provides the full results.

**Table 2.** Full interview findings

Category		Statement and number of participants who express a view^a^	Stakeholder group
*The General Strengths and Challenges of the EUnetHTA initiative and JA 3*			
The intent of the EUnetHTA initiative	Likert Scale question: I regard the intent of the EUnetHTA initiative as positive	Mean 4.5	In total
		Standard deviation 0.6	In total
		Value of 1 (Full refusal of the statement) (*n*=0)	—
		Value of 2 (Rather a refusal of the statement) (*n*=0)	—
		Value of 3 (Neutral to the statement) (*n*=1)	HTAb
		Value of 4 (Rather an agreement with the statement) (*n*=7)	A, HTAb, P, PO
		Value of 5 (Full agreement with the statement) (*n*=10)	A, EUn, HTAb, PO, R
The outcome of the EUnetHTA initiative	Likert Scale question: I regard the outcome of JA 3 of the EUnetHTA initiative as positive	Mean 3.2	In total
		Standard deviation 0.7	In total
		Value of 1 (Full refusal of the statement) (*n*=1)	A
		Value of 2 (Rather a refusal of the statement) (*n*=0)	—
		Value of 3 (Neutral to the statement) (*n*=11)	A, EUn, HTAb, P, PO, R
		Value of 4 (Rather an agreement with the statement) (*n*=6)	A, EUn, HTAb, P
		Value of 5 (Full agreement with the statement) (*n*=0)	—
JA 3	Strengths	Consolidation of the foundation of information exchange (thus, a common view toward the challenges and an increased understanding of HTA requirements of drugs’ clinical effectiveness) (8)	A, EUn, HTAb, P, PO
		The high quality of the published documents (guidelines, reports) (7)	A, EUn, HTAb, PO, R
		Clinical data within Europe should in principle, apply first because of the similarity of the population (2)	EUn, P
		Example for other continents of how collaboration could happen (1)	EUn
		Increased understanding of the HTA community towards the mechanisms behind the benefit-risk ratio (1)	A
		Establishment of a strong synergy with the EMA with its strong expertise in, e.g., patient involvement (1)	PO
		Learning that a systematic and more centralized approach of stakeholder involvement is needed for every activity (1)	PO
		Proof of ability to implement common plans at the scientific and technical level (1)	EUn
	Challenges	The difficulties of navigating as a multi-state with decision-making, e.g., in coming to agree on the scope of collaboration vs. the perceived risk to the sovereignty of the HTAbs (7)	A, EUn, HTAb, P, PO, R
		Difficulties of agreeing on joint methodologies (7)	A, HTAb, P, PO, R
		Challenges related to the legislative legitimacy (7)	A, EUn, HTAb, P, PO, R
		The JAs were time-limited, making it difficult to familiarize with and constantly adapt to procedures (5)	A, EUn, HTAb, PO, R
		Challenges pertaining to tangible efficiencies of process (5)	A, HTAb, P
		Varying strengths of the learning effect of the HTAbs (some gained the HTA experiences that others already had) (4)	EUn, HTAb, P, R
		Challenges related to the funding from the EU (3)	Eun, R
		Ensure patient impact in HTA as there is no evidence that the patient experience has impacted the decision-making in HTA, e.g., through more involvement and the provision of training for patients on HTA (3)	P, PO
		Lack of completed projects (rapid REAs or EDs) (2)	A, PO
		With regard to proposed future legislation, the liability of not being able to request further data from the pharmaceutical industry for the national assessment (1)	A
		Challenges related to the constant awareness of the national HTAb about collaboration between HTAb on the European level to leverage developed synergies and use them at a national level for outcomes on the decision-making (1)	PO
*The Strengths and Challenges in the Clinically oriented HTA Collaboration in Oncology during JA 3*			
Horizon scanning	Strengths	The pilot project performed was an adequate first approach for the timely initiation of joint assessments (5)	A, HTAb, P, R
		The priority list was a basis for approaching the manufacturer of the selected drug with the topic of implementing a rapid REA for this drug (1)	HTAb
	Challenges	The non-existence of a collaborative list on which technologies will be assessed jointly (2)	HTAb
		Timing-related challenges (1)	HTAb
		Priority-related open points and issues (a political decision about prioritized drugs could mitigate this) (1)	HTAb
		Some manufacturers’ non-acceptance of the inclusion of a specific drug into the rapid REA process led to challenges in the transition from HS to rapid REA (1)	HTAb
	Other	The regulation on HTA can provide guidance on which drugs should be assessed jointly (1)	HTAb
Early dialogue	Strengths	Allowing for the development of the existing common positions within the PICO scheme (like the consensus towards the endpoints, the segmentation of the study population, tumor-agnostic therapies) and for jointly foreseeing potential evidence gaps/major or minor issues that could occur in the national assessments of new modes of action with the expected added benefit (8)	A, EUn, HTAb, P, R
	Challenges	Patient involvement: The lack of systematic involvement of the patients (2) (e.g., inconsistent sharing of the briefing book, participation in the ED meeting, and access to the final advice (1))	PO
		Constrained resources (2)	HTAb, P
		The non-binding character reduces the ED’s helpfulness in preparing for the assessments (2)	P
		The time gap between the ED and the assessment (1)	P
Rapid relative effectiveness assessment	Strengths	The relatively high content-wise applicability of the reports (PICOT, for instance, the comparator) for national reporting requirements (mentioned for the efficacy and safety data) for national contextualization (7)	A, EUn, HTAb
		The scientific quality (4)	A, HTAb
		The scoping phase included the view of the HTAbs and/or the patient organizations (3)	HTAb, PO
		The time-saving potential of the reports due to their clear structure (3)	A, HTAb
		The transparency of the delivery process or the content of the report (2)	A, HTAb
	Challenges	Reaching a consensus on the clinical evidence requirements regarding standard of care, and/or the comparators/indirect comparisons and/or the acceptance of (patient-relevant or surrogate) endpoints between the HTAbs and/or the different opinions on what (size and non-RCT) evidence and analyses to include (10)	A, HTAb, P, PO, R
		Lack of enough rapid REAs on relevant products (5), referring to the low number of reports (2), challenges to including relevant compounds for rapid REAs throughout the industry due to the industry’s uncertainty that comes with a new process (2), the low budget impact of the assessed drug (1)	A, HTAb, PO
		The complexity of the document is too high (4)	A, EUn, HTAb
		Lack of a legal mandate to have joint assessments (4)	A, EUn, P
		Timing-related challenges (availability of the final report) (3)	A, HTAb
		Varying adoption of the reports (3)	A, EUn
		The creation of the reports is time-consuming for the authoring teams and further stakeholders involved (3): -the scoping phase and review rounds increase the duration of the process (2) -the variability of the authoring teams led to inefficiencies	HTAb
		Some HTAbs are concerned that joint rapid REAs might reduce the influence of the HTAb in the final decision (2)	A, EUn
		Specific evaluation regarding the occurrence of side effects or health-related impact on quality of life is not included (1)	HTAb
		The impact of oncology on the budget might increase caution towards the adoption of rapid REAs from other agencies (1)	EUn
		Lack of an integrated approach with patient involvement post-scoping and a clear framework about the impact of patients’ input on the reports (1)	PO
		Interactive patient involvement post-scoping (1)	PO
		How much weight certain HTAs put into patient preferences or quality of life assessments (1)	R
		The lower added value of the reports in the case of non-extensive need for literature research, indirect comparators, or any comparators (1)	HTAb
		The legal obligation to submit own reports (1)	HTAb
		A better sharing of information between regulator and HTAbs during the regulatory assessments (1)	R
Postlaunch evidence generation	Strengths	Pilots (for joint indication-based registries) were conducted (2)	HTAb
	Challenges	More collaboration is needed for PLEG than for the activities or milestones that took place (5)	A, EUn, HTAb
		Differences in the technical and personnel resources, data infrastructure, or experience to evaluate and collect the relevant data (2) but some countries could share their gathered data (1)	HTAb, R
		Aligning endpoint requirements between EMA’s PASS and PAES with HTA requirements and the lack of a legal option for imposing the generation of further evidence (1)	A
		Restrictions in the accessibility of the data (the confidentiality and laws around the registries) (1)	HTAb
*The Future of HTA in Oncology*			
The expected upcoming developments and challenges for HTA in oncology		Increasing uncertainty towards the HTAb (single-arm trials, smaller subpopulations, surrogate endpoints, conditional and exceptional marketing approvals) (8)	A, EUn, HTAb, P, PO, R
		The rapid evolution of many new therapeutic innovations (for instance, the identification of biomarkers and genome defects, Advanced Therapy Medicinal Products, upcoming innovations) (7)	A, EUn, HTAb, P, R
		Challenges in affordability (4)	EUn, HTAb
		Developing instruments to measure the quality of life or Patient-Reported Outcomes while including the specificity of a specific indication (3)	A, EUn, PO
		The evolution of national outcome-based agreements like pay for performance approaches might be increasingly combined with registries (3)	A, EUn, HTAb
		No standard methodology for assessing new interventions is in place yet (2)	EUn
		Innovative, digital ways to perform research and development (1)	EUn
		Orphan Drugs being approved for multiple indications (1)	A
Expected developments regarding collaboration in the clinical domains of HTA		Generally referring to the new regulation and the arrangements that remain to be seen (9)	A, EUn, HTAb, P, PO, R
		The persistent need to avoid double work leads to an increasing demand for collaboration (7)	A, EUn, HTAb, P
		Need for the scientific, methodological evolution of HTA (7), specifically, jointly finding new ways to deal with or accept the uncertainty when assessing oncology drugs (2) or the opportunity to agree on joint criteria for evaluating minor absolute effects (1)	EUn, HTAb, P, PO
		Call for joint PLEG activities in suitable cases to acquire more data and define ways how to deal with the data (5)	EUn, HTAb, P
		Not necessarily an increasing demand for collaboration due to the workload of the assessments (rather a question of collaborative potential when it comes to the acceptance of uncertainty) (2)	EUn, HTAb
		The potential to collaborate in the reassessment of existing and renumerated drugs, e.g., through sharing of the reassessment signals (2)	HTAb, R
		Collaboration through patient involvement is relevant for all diseases, and collaboration is also relevant for those with low patient populations, to obtain enough input from patients (1)	PO
		Regulator and HTA collaboration in systematically incorporating patient preferences and quality of life data in the assessment of medicines or through the regulator’s provision of a 3-year forecast to the HTAbs on which sort of medicine they are going to receive (1)	R
		The benefit of further joint HS activities (1)	HTAb
		The organizational structure of the companies will adapt, depending on the binding nature of a JCA. (1)	P
		HTA-related discussions about a scale with different levels of unmet need will be needed in the future; this might consider or be limited by the absolute effect of a drug regarding, e.g., overall survival (1)	EUn
		The uncertainty about assessing benefits may hamper cooperation between the HTAbs (1)	HTAb
		Collaboration in rapid REAs with existing standard operating procedures (1)	EUn
*The Potential of a Voluntary Cooperation in Nonclinical Domains in Oncology*			
Collaboration in general		Different economic backgrounds, national legislation, decisions on how to spend their money, or different dates of availability of oncology drugs need to be considered (11)	A, EUn, HTAb, P, PO, R
		Provision of examples of joint payer negotiations from the interviewees referring to a more substantial negotiation power (even though outside the scope of HTA, they demonstrate that collaboration on drugs’ market access can go beyond the clinical area of HTA) (5)	A, EUn, HTAb, P
		Critical for further developments is whether nonclinical cooperation would be voluntary or partly mandatory (2)	A, R
		It would benefit the HTA community if every HTAb would share their approaches, e.g., through publishing the reports in a structured form (2)	HTAb, PO
		An agreement of methods for nonclinical HTA would benefit the HTA community (1)	PO
		BeNeLuxA is an example of collaboration in economic evaluation (1)	A
		Managed entry agreements could, in principle, take the form of a multi HTA-oriented collaboration (1)	P
The potential clustering of individual EUnetHTA efforts into the concepts of added value versus cost-effectiveness/cost-utility	Likert Scale question: I regard the clustering into the concepts of added value versus cost-effectiveness/cost-utility as feasible	Mean 3.2	In total
		Standard deviation 1.1	In total
		Value of 1 (Full refusal of the statement) (*n*=1)	HTAb
		Value of 2 (Rather a refusal of the statement) (*n*=3)	HTAb, EUn, A
		Value of 3 (Neutral to the statement) (*n*=8)	P, R, A, HTAb, PO
		Value of 4 (Rather an agreement with the statement) (*n*=3)	A, PO, HTAb
		Value of 5 (Full agreement with the statement) (*n*=3)	P, EUn, HTAb
	Opportunities related to the CEA/CUA	The collection of data or shared literature searches related to quality- or disease adjusted life-years (excluding national conversion) (3)	HTAb
		The EDs hold further potential for systematizing nonclinical collaboration, and the countries that perform a CEA already use the EDs to coordinate their needs for the CEA (1)	HTAb
		Potentially, joint cost-utility analyses in the distant future (1)	P
	Opportunities related to the AV	The similar structure of scales could be a starting point (3)	A, HTAb
		The similar decision towards the height of reimbursement for oncology drugs in the past (between France and Germany) (2)	A, P
	Other opportunities	Learning from other countries what they perceive as necessary steps and taking these aspects into account for their own economic evaluation (1)	HTAb
		Closer collaboration between the regional initiatives and European-wide initiatives (1)	HTAb
		This approach provides the opportunity to steer the discussion on collaboration in the direction of the similarities which already exist. (1)	P
		The industry’s new product planning framework already functions through gathering data requirements for these two different concepts (1)	P
		There might be completely new forms of collaborative initiatives in economic evaluation (1)	EUn
	Challenges related to the CEA/CUA	At least the conclusion of the CEA cannot be the same for the countries within the clusters (4)	A, HTAb, P
		Differences in the cost information of medical resources (2)	HTAb
		Differences in quality of life-related aspects (1)	HTAb
	Challenges related to the AV	No need for alignment as the system that is completely tailored towards the national needs is working properly (1)	HTAb
		Different decisions towards the level of the assigned added value for oncology drugs in the past (between France and Germany) (1)	A
	Other challenges	Due to the many and significant differences between the countries’ nonclinical HTA, there would be lower efficiency savings for the health systems in case of collaborative efforts in nonclinical domains than for the clinical fields (1)	R
		Differences in the population (1)	HTAb
		The different timing of price negotiations between the countries (1)	A

a Alternatively, the mean or standard deviation of the evaluation of the Likert Scale question.

A, academia; AV, added value; BeNeLuxA, HTA collaboration between Belgium, the Netherlands, Luxemburg, Austria, and Ireland; CEA, cost-effectiveness analysis; CUA, cost-utility analysis; ED, early dialogue; EMA, European Medicines Agency; EU, European Union; EUnetHTA, European Network for Health Technology Assessment; EUn, EUnetHTA senior board; HS, horizon scanning; HTA, health technology assessment; HTAb, HTA body; JA 3, Joint Action 3; JCA, joint clinical assessment; M, mean; P, pharmaceutical industry; PAES, post-authorization efficacy study; PASS, post-authorization safety study; PICO, population, intervention, comparison, outcome; PLEG, postlaunch evidence generation; PO, patient organization; R, regulator; rapid REA, rapid relative effectiveness assessment; RCT, randomized controlled trial; SD, standard deviation.

### The strengths and challenges of the EUnetHTA’s JA 3

On the one hand, the EUnetHTA’s JA 3 initiative consolidated the foundation of information exchange, consequently allowing for a common view of HTA, in particular with regard to the requirements for the clinical effectiveness of a drug (8: A, EUn, HTAb, P, PO). In addition, the guidelines and reports produced were of high quality (7: A, EUn, HTAb, PO, R). On the other hand, on average, stakeholders perceived the outcome of JA 3 as neutral. Specifically, the initiative’s challenges often featured multi-state navigating through decision-making to agree on the scope of collaboration (7: A, EUn HTAb, P, PO, R). Moreover, the legislative legitimacy of the EUnetHTA’s projects was not always provided (7: A, EUn, HTAb, P, PO, R), leading to uncertainty among stakeholders about the initiative’s future.

### The clinically oriented HTA collaboration during JA 3

This section looks at collaboration during JA 3 more broadly. Specific areas of collaboration included in the interviews are HS, EDs, rapid REAs, and PLEG. HS should lead to a central overview of all forthcoming approved drugs and the joint prioritization of those drugs which are to be assessed during rapid REAs. Even though complete related lists or rigorous decision structures such as who should perform the joint prioritization were not in place (4: HTAb), the experts valued the EUnetHTA’s HS pilot project as an important first step toward identifying the critical elements for successful HS activities (5: A, HTAb, P, R). It was challenging to foresee all potentially available drugs, as the timing between HS activities and the provision of relevant information from the EMA were not aligned from the beginning (1: HTAb). The challenges in the EUnetHTA’s legislative legitimacy (7: R, PO, A, HTAb, P, EUn) might have led to EMA’s prohibition against providing relevant information to them. Based on the HS decisions, EUnetHTA stakeholders asked manufacturers if their drug could be subjected to rapid REA. In individual cases, some companies’ refusal made it necessary to adjust the process (1: HTAb).

EDs also allowed for the development of cross-stakeholder positions and opened the possibility to jointly foresee potential gaps in evidence that could occur in the national assessments of new modes of action (8: A, EUn, HTAb, P, R). However, the non-binding character and time gap between an ED and the actual assessment reduced their applicability to industry (2: P). In addition, constraints on resources and the resulting need to select which candidates could undergo EDs posed a challenge (2: HTAb, P). The consistent impact of patients’ involvement on the EDs’ outcomes was questionable (2: PO), as, for example, sharing ED-related information with patients and their inclusion in the EDs’ meetings were not consistently ensured (1: PO). Regarding the overall context of EDs, an interviewee from the EUnetHTA proposed that “EDs should be the starting point for an overall evidence generation plan, including the different needs of the HTAbs and thus going beyond the design of […] phase three clinical trial.”

The rapid REAs formed the core of the cooperation. There were only a limited number of rapid REAs, but they were of high quality and allowed for efficient contextualization to national requirements (7: A, EUn, HTAb). However, the authoring HTAbs were confronted with methodological challenges during the development process, including differences in standards of care, the handling of comparators and endpoints, and the inclusion of evidence from a non-randomized controlled trial (non-RCT) (10: A, HTAb, P, PO, R). Some participants also stated that the adoption of the reports varied between the drugs and HTAbs (3: A, EUn), and viewed the timing of the final report’s availability as a challenge, especially for countries that need to assess each drug within a specific timeframe after market approval (3: A, HTAb). Specifically, the pre-assessment’s scoping phase and review rounds were named as drivers of the duration of the process (2: HTAb). During the scoping phase, each stakeholder participating in the EUnetHTA was allowed to provide input (including population, intervention, comparison, and outcome) for a specific framework before the authoring HTAbs conducted their reports. Moreover, the constellations of HTA authors varied, leading to a diversity of views and exchange but also to process inefficiencies (1: HTAb). One stakeholder from the patient organizations missed an integrated framework that shows the specific impact of the input from patients on the reports.

Several participants indicated further that collaboration on PLEG should be expanded (5: A, EUn, HTAb). Specific hurdles included differences in resources, data infrastructure, and accessibility, as well as varying levels of experience with the synthesis of evidence (2: EUn, R). Data sharing from more experienced countries could support further development, while ways to manage confidentiality need to be initiated (1: HTAb). Despite these challenges, and in coordination with the existing legitimacy and experience of the regulator to impose the generation of new evidence, the PLEG requirements between the regulator and HTAbs should harmonize (1: A).

### The future of HTA

The participants expect to see more single-arm trials with smaller subpopulations and surrogate endpoints in the future, leading increasingly to conditional or exceptional marketing approvals (8: A, EUn, HTAb, P, PO, R). Scientific progress manifested in personalized medicines targeting specific genetic expressions will provide potentially long-standing treatments or cures. It can be challenging, using current HTA methodologies, to assess these innovative medicines – for instance, to measure overall survival. As a result, for some stakeholders, there is a growing need for cooperation in clinically oriented HTA for developing new methodologies (7: EUn, HTAb, P, PO). Moreover, joint PLEG activities should be developed further. However, some of the experts’ expectations for future HTA collaboration, such as rapid REA reports and increasing permanent collaboration, will be addressed through the Regulation of the European Parliament and the Council on Health Technology Assessment (9: EUn, HTAb, P, PO) (10).

In the economic domains of HTA (3), existing differences, including economic contexts and the costs or resources of the medical sector, are barriers to collaboration (11: A, EUn, HTAb, P, PO, R). Nevertheless, the stakeholders proposed some areas of voluntary collaboration. Within HTA, these include the publication of nonclinical reports (2: HTAb, PO), and joint activities related to instruments for measuring quality-adjusted life years or disability-adjusted life years (3: HTAb). The BeNeLuxA initiative members already share information regarding nonclinical elements (1: A). The context of voluntary yet clinically oriented EDs also has potential for systematizing nonclinical collaboration.

## Discussion

This study provides insights into past and future HTA cooperation. It suggests that different stakeholders perceive the EUnetHTA’s various collaboration activities as valuable. However, on average, stakeholders rate the outcome of JA 3 as neutral. The participants cite challenges in HTA cooperation until 2021 and suggest possibilities for future collaboration. Joint work in HS systems and PLEG is still in the early pilot phase, but has the potential to mitigate uncertainty in the emergence of new technologies or gaps in evidence. EDs also help anticipate potential uncertainty, especially regarding a drug’s clinical effectiveness, and allow for international consensus on study design requirements. According to the stakeholders, until 2021 the main barriers to HTAbs’ adoption of rapid REAs included an insufficient number of relevant reports, different perspectives on comparators and endpoints, the inclusion of non-RCT evidence, and timing variances. A joint overall plan to generate evidence across the technology life cycle is proposed for clinically oriented HTA collaboration. The participants regard voluntary joint work in nonclinical HTA as challenging, but provide existing isolated approaches in countries with similar methods.

These findings are relevant for policymaking, especially as, to the authors’ knowledge, this is the first study that includes multi-stakeholder views on collaboration in clinical, nonclinical, and oncology domains. The coordination group and its subgroups can use these results when preparing to implement the HTA regulation (10). The coordination group consists of delegated members of the European member states. It provides the strategic direction for the work of its subgroups. The subgroups are composed of national or regional authorities and work on specific collaborative topics (10). First, under the new regulation, EUnetHTA EDs, referred to in the future as joint scientific consultations (JSCs) (10), are performed only for selected drugs. Applying JSCs to all potentially innovative drugs would strengthen HTA collaboration. The EUnetHTA also recommends a life cycle approach by sharing information from the JSC assessment teams, while remaining evidence gaps identified in assessments should be referred to PLEG activities (4). The interviewees valued PLEG’s opportunity to mitigate the uncertainties related to increasingly conditional or exceptional marketing approval, which were also highlighted by Moseley et al. (12). The HTA regulations contain a reference to voluntary cooperation in real-world evidence and to supporting the further development of related databases and registries (10). It will be of interest for all stakeholders to monitor the options for joint PLEG that will arise at the European level. Second, sufficient capacity must be made available to create the JCAs so that countries can meet their national timelines. These reports should include a description of the relative effectiveness and analysis of scientific uncertainties, but no binding conclusion (10). Thus, third, the reports’ content and structure will allow for practical contextualization to account for the different evidence requirements described in this and other studies (13;14). Overall, the transparency and documentation of all HTA-related, non-confidential information at the EU level to countries and vice versa will be essential to further implement and develop collaboration.

This study also provides insights to plan optional collaboration in nonclinical domains. The participating experts perceived boundaries as strong, mainly due to differences in national methodologies. However, there are European methodological recommendations on economic evaluation (15;16). The experts also mentioned jointly collecting data or developing instruments to measure quality-adjusted life years or disability-adjusted life years. The instruments required to measure health status, such as the preference-based EQ-5D questionnaire, are present across Europe (17).

According to this research, patient organizations do not yet feel that the patient’s perspective is systematically involved in HTA collaboration. At the same time, there are already frameworks outlining relevant criteria for their involvement (18;19). A further study also claimed that patient involvement should be further strengthened (20). Ways toward the more impactful and systematic involvement of patient organizations in HTA could be a subject for further investigations.

There are some limitations to this study. To start, despite the initial focus on oncology, the results are mostly not oncology-specific. To obtain results that are truly oncology-focused, it may have been necessary to include examples of oncology drugs in the interviews. Still, the study’s general conclusions are of broad relevance and suggest that procedural and methodological challenges to HTA collaboration do not necessarily differ between therapeutic areas. Another study limitation is that the insights generated in the eighteen interviews do not create a representative picture, especially for stakeholders other than HTAbs. Moreover, the HTAbs came from Northern European countries and further research would be needed with other agencies participating in the EUnetHTA to mitigate potential selection bias. Given time constraints, one researcher did the data analysis, whereas several researchers could have better underlined and differentiated the findings.

## Conclusion

This study delivers present and future-oriented insights into European HTA cooperation. The stakeholders involved support clinically oriented collaboration across the technology life cycle to cope with the uncertainty of relative effects between drugs. They also indicate that forthcoming collaboration on HTA must allow for practical content- and timing-related contextualization of the drug’s relative effectiveness, as differences remain in the requirements for evidence. There is potential for future collaboration in the PLEG field, but also severe challenges. Further, patients still need to be involved more systematically in European HTA. In the nonclinical area of HTA, countries using cost-effectiveness or cost-utility analysis could cooperate with each other more closely.
